# Dental derived stem cell conditioned media for hair growth stimulation

**DOI:** 10.1371/journal.pone.0216003

**Published:** 2019-05-01

**Authors:** Tarini Nawamalie Abeysinghe Gunawardena, Zeinab Masoudian, Mohammad Tariqur Rahman, Thamil Selvee Ramasamy, Anand Ramanathan, Noor Hayaty Abu Kasim

**Affiliations:** 1 Department of Restorative Dentistry, Faculty of Dentistry, University of Malaya, Kuala Lumpur, Malaysia; 2 Stem Cell Biology Laboratory, Department of Molecular Medicine, Faculty of Medicine, University of Malaya, Kuala Lumpur, Malaysia; 3 Department of Oral & Maxillofacial Clinical Sciences, Faculty of Dentistry, University of Malaya, Kuala Lumpur, Malaysia; 4 Oral Cancer Research & Coordinating Centre (OCRCC), Faculty of Dentistry, University of Malaya, Kuala Lumpur, Malaysia; 5 Regenerative Dentistry Research Group, Faculty of Dentistry, University of Malaya, Kuala Lumpur, Malaysia; Università degli Studi della Campania, ITALY

## Abstract

Alopecia is a clinical condition caused by excessive hair loss which may result in baldness, the causes of which still remain elusive. Conditioned media (CM) from stem cells shows promise in regenerative medicine. Our aim was to evaluate the potential CM of dental pulp stem cells obtained from human deciduous teeth (SHED-CM) to stimulate hair growth under *in vitro* and *in vivo* conditions. SHED and hair follicle stem cells (HFSCs) (n = 3) were cultured in media combinations; i) STK2, ii) DMEM-KO+10% FBS, iii) STK2+2% FBS and profiled for the presence of positive hair growth-regulatory paracrine factors; SDF-1, HGF, VEGF-A, PDGF-BB and negative hair growth-regulatory paracrine factors; IL-1α, IL-1β, TGF-β, bFGF, TNF-α, and BDNF. The potential of CM from both cell sources to stimulate hair growth was evaluated based on the paracrine profile and measured dynamics of hair growth under *in vitro* conditions. The administration of CM media to telogen-staged synchronized 7-week old C3H/HeN female mice was carried out to study the potential of the CM to stimulate hair growth *in vivo*. SHED and HFSCs cultured in STK2 based media showed a shorter population doubling time, higher viability and better maintenance of MSC characteristics in comparison to cells cultured in DMEM-KO media. STK2 based CM contained only two negative hair growth-regulatory factors; TNF-α, IL-1 while DMEM-KO CM contained all negative hair growth-regulatory factors. The *in vitro* study confirmed that treatment with STK2 based media CM from passage 3 SHED and HFSCs resulted in a significantly higher number of anagen-staged hair follicles (p<0.05) and a significantly lower number of telogen-staged hair follicles (p<0.05). Administration of SHED-CM to C3H/HeN mice resulted in a significantly faster stimulation of hair growth in comparison to HFSC-CM (p<0.05), while the duration taken for complete hair coverage was similar for both CM sources. Thus, SHED-CM carries the potential to stimulate hair growth which can be used as a treatment tool for alopecia.

## Introduction

Hair loss has a major impact on the social interactions and psychological well-being of an individual [1], as appearance plays a critical role in non-verbal communication [2]. The condition of hair loss from the head or body in clinical terms is referred as “alopecia”, which may eventually result in baldness [3].

The current treatment for alopecia is the use of Finastride and Minoxidil [4]. Although proven to be effective, discontinuation of these drugs carries the risk of accelerating hair loss. An alternative approach, alopecia surgery can only be performed on an individual for a maximum of 3 times and the number of hair strands that could be transplanted during each surgical procedure is limited to a maximum of 2000 [3]. Thus, effective treatment strategies are yet to be developed, in order to overcome the issues that are faced by the current treatment strategies.

Hair growth is a cyclic process categorized into three main stages; anagen (active phase), catagen (resting phase) and telogen (regression phase). In all four major forms of alopecia; androgenic alopecia, telogen effluvium, chemotherapy-induced alopecia and alopecia areata, the hair follicles at the anagen stage enter the catagen or telogen stages simultaneously resulting in early shedding of hair. Thus, the treatment strategy for hair loss involves an approach of prolonging the anagen phase of the hair cycle and reducing the number of hair follicles that are in the telogen stage. However, it is pertinent to note that the latter two forms of alopecia are generally reversible conditions since the hair follicle stem cells (HFSCs) are not affected.

Currently, HFSCs obtained from scalp biopsies have been expanded under *in vitro* culture conditions and injected into the bald scalp regions for the stimulation of hair growth [5]. Ongoing phase II clinical trials are carried out by using adipose-derived stem cells (ADSCs) [6] and autologous dermal cells [7] for the stimulation of hair growth. However, cell-based therapies for tissue regeneration present a number of challenges such as low survival rate of transplanted cells, immunological responses in the event of post-administration, reduced regenerative potential (proliferation and differentiation) of transplanted cells, lower treatment efficiency due to the presence of heterogeneous population, increased oncogenic potential of the genetically manipulated cells and cells acquiring a cancer cell-like behaviour when administered to normal tissues [8–13]. To overcome these shortcomings, the regenerative induction by stem cells is harnessed through a novel approach of administration of the paracrine factors secreted by these cells.

Paracrine factors play an important role in stimulation of the molecular and cellular processes that govern hair growth. Recent studies have demonstrated that paracrine factors EGF, TGF-β, IGF-1, HGF, VEGF [14] and FGF-10 are responsible for the regulation of human hair growth. Additionally, TGF-α, aFGF, bFGF, FGF-5 and PTHrP are known to regulate the hair growth in animal models such as sheep and mice [15–16]. Thus, conditioned media (CM), a cell-free, paracrine factors rich source [10] could be an alternative to cell-based therapies.

Multipotent HFSCs present in the bulge region are shown to exhibit neural crest characteristics. Similarly, teeth is an ectodermal organ which originates from interactions between the cranial neural crest and oral epithelial cells [17]. Dental pulp stem cells (DPSCs) have been demonstrated to possess the ability to differentiate into hair follicles, neural cells, elastic cartilage cells, skeletal and/or smooth muscle cells, endothelial cells, adipocytes, osteoblasts, dentine producing odontoblasts under *in vitro* and *in vivo* conditions [18–19]. Even though DPSCs have been shown to have a regenerative potential for the generation of hair follicles [20], further studies are required to identify culture conditions that influence the secretion of hair stimulating factors and prepare these stem cells for transplantation that warrant its application to treat alopecia. Compared to many other sources, dental pulp tissues yield a relatively higher number of stem cells [21]. The paracrine factor profiles of these cells indicate the presence of many positive hair growth-regulatory factors [22].

Thus, based on the capability of DPSCs to regenerate hair follicles [20], and their paracrine secretion profiles, we aimed to investigate the hair growth potential of CM of dental pulp stem cells obtained from human deciduous teeth (SHED) in comparison to CM produced using HFSCs.

## Methodology

### Cell culturing of SHED and HFSCs

SHED and HFSCs Cells at Passage 1 were purchased from AllCells, USA and ICELLTIS, Labege, France respectively. The cells were expanded at a seeding density of 5000 cells/cm^2^ in different media combinations; (i) DMEM-KO (Gibco Invitrogen, Carlsbad, CA, USA) + 10% FBS (Gibco Invitrogen, Carlsbad, CA, USA) +1% 4 mM Glutamax (Gibco Invitrogen, Carlsbad, CA, USA) + 0.5% pen-strep (Gibco Invitrogen, Carlsbad, CA, USA), (ii) STK2 (TwoCELLS, Japan) + 2% FBS+ 1% 4 mM Glutamax+ 0.5% pen-strep and (iii) STK2 + 1% 4 mM Glutamax+ 0.5% pen-strep and maintained at 37°C and 5% CO_2_.

### Growth kinetics of SHED and HFSCs

Sub-culturing of passage 1 HFSCs and SHED to passages 2–5 was carried out upon 80% confluency. The viability assessment of the cells for each passage was carried out by Trypan blue dye (Gibco Invitrogen, Carlsbad, CA, USA) exclusion method using Countess Automated cell counter (Gibco Invitrogen, Carlsbad, CA, USA). Readings for the total, live and dead cell counts, and the viable cell percentage were obtained.

The population doubling time (PDT) for each sample was measured using the following formula [23].

$$PDT=\frac{\Delta t}{\log_{2}\left\{(\frac{\Delta N}{N0})+1\right\}}$$

PDT = population doubling time

Δt = time taken for 80% confluency

ΔN = difference in cell number

N_0_ = total cell number seeded

### Characterization of SHED and HFSCs

The mesenchymal stem cell (MSC) properties of the cells were characterized, as per the guidelines specified by the International Society for Cellular Therapy [24]. Passage 3 SHED and HFSCs were seeded at a density of 5000 cells/cm^2^. Upon 80% confluency, 2 ml each of adipogenic, chondrogenic, osteogenic differentiation media (ThermoFisher Scientific, Waltham, MA, USA) and the corresponding culture media was added to each well and media change for the corresponding differentiation lineage was carried out every 3 days. Staining was conducted for adipogenic (Oil red O (Sigma-Aldrich, Missouri, USA)) lineage at day 14, chondrogenic lineage (safranin (Sigma-Aldrich, Missouri, USA)) at day 28 and osteogenic (Alzarin Red (Sigma-Aldrich, Missouri, USA)) lineage at day 21 for HFSCs and day 22 for SHED, and the cells were observed under Olympus CKX41 inverted microscope (Olympus, Tokyo, Japan).

Flow-cytometry analysis was conducted for 80% confluent, passage 3 SHED and HFSCs to determine the expression of positive mesenchymal stem cell markers CD90, CD105 and CD73, and negative mesenchymal stem cell markers CD45, CD34, CD14, CD11b, CD79α, CD19 and HLA-DR.

### Collection and characterization of conditioned media

SHED-CM and HFSC-CM was prepared by supplementing the cells with fresh STK2 or DMEM-KO upon 80% confluency and incubated for 24 h. The CM was collected in pre-chilled tubes followed by centrifugation at 310 g for 6 min at 4°C. The media was filtered using 2 μm filters (Thermo Fisher Scientific, MA, USA) and collected in pre-chilled 1.5 ml microcentrifuge tubes (Eppendorf, Hauppauge, NY, USA).

The CM was assayed using the Luminex (Affymetrix, Ebioscience, USA) with a customized Procata (Affymetrix, Ebioscience, USA) Human 10-plex panel comprising the analytes SDF-1, HGF, VEGF-A, PDGF-BB, IL-1α, IL-1β, TGF**-**β, bFGF, TNF-α and BDNF. The concentration of each analyte was measured using the Luminex system in combination with the ProcataPlex Analyst version 1.0 software using four parametric curve fitting.

### Analysis of *in vitro* hair growth stimulation by conditioned media

All animal experimental protocols were carried out following the ethics approval obtained from the Animal Ethics Committee of University of Malaya (2015-180407/RESTD/R/NHAK).

Seven-week-old female ICR mice were purchased from the Animal Experimental Unit, Faculty of Medicine, University of Malaya. Following the euthanization of mice in CO_2_ euthanization chamber for rodents, the dorsal region of the mice was shaved with clippers, and 0.5 cm x 0.5 cm skin samples from the shaved area was cut till the subcutaneous region. Three cut of skin pieces measuring approximately 0.5 cm x 0.5 cm were supplemented with 300 μl of CM and incubated at 37°C for 72 h. At every 24 h time point one skin sample from each well was processed for histological analysis by fixing in 10% of formaline (Sigma-Aldrich, Missouri, USA) for 48 h. The skin tissues were positioned vertically and embedded in paraffin wax. The tissues were sectioned using the Leica Microtome (Leica, Wetzlar, Germany) longitudinally to obtain 5 μm sections followed by heamatoxylin and eosin (Sigma-Aldrich, Missouri, USA) (H&E) staining. The tissues were scanned using the digital scanning system (Pannoramic Desk 3DHISTECH, Budapest, Hungary) and the micrographs were read using the Pannoramic viewer software (3DHISTECH, Budapest, Hungary).

The stage of murine hair growth was identified using a scoring chart (S1 Fig) adapted as per guidelines set by Muller-Rover et. Al [25] and scored by two observers (TNAG and AR) and the inter-reliability of scoring was calibrated against each other. The percentage of hair follicles present in the anagen, telogen and catagen stages were tabulated. Students paired t-test (SPSS Version 22) was carried out to ascertain the differences between the three CM in supporting the transition of hair growth to the different stages, within a period of three days, at the confidence level of 95%.

### Analysis of *in vivo* hair growth stimulation by conditioned media

Five week old C3H/HeN female mice (n = 20) was purchased from M-Clea Bioresources Co., Ltd., Thailand. The mice were acclimatized under controlled environmental conditions (room temperature 24 ± 1°C, humidity 65–75%) for 14 days. The mice were fed on an altromin diet, comprised of crude protein (18.5% per kg), fat (6.2% per kg), fiber (5.6% per kg), ash (7.2% per kg), moisture (11.3% per kg) and nitrogen free extract (51% per kg).

Upon seven weeks of age the mice were anesthetized using ketamine, xylasine intraperitoneal and shaved with clippers. The pink coloured skin observed upon shaving, indicated that the hair growth stage is now synchronized to telogen stage [25]. The mice were divided into four groups; Group 1- mice administered with SHED-CM (n = 9), group 2- mice administered with HFSC-CM (n = 9), group 3- mice administered with STK2 media (n = 3), group 4- mice with no treatment (n = 2), where in groups 3 and 4 are the control groups.

Each mouse in groups 1, 2 and 3 were administered with 3 sub-cutaneous injections of 100 μl of the selected media at the dorsal region at 3-day intervals and the darkening of skin was monitored pictorially on alternate days to observe the hair growth progression. The mice were euthanized using CO_2_ gas in the euthanization chamber for rodents when almost complete hair growth was observed in the shaved area. The Mann-Whitney U test was chosen to determine the differences between all groups in stimulating the anagen stage of hair growth at the confidence level of 95%.

### *In vivo* toxicity studies of the conditioned media

Upon seven weeks of age, n = 16 C3H/HeN mice were divided into four groups; Group 1- mice administered with SHED-CM (n = 4), group 2- mice administered with HFSC-CM (n = 4), group 3- mice administered with STK2 media (n = 4), group 4- mice with no treatment (n = 4). Each mouse in groups 1, 2 and 3 were administered with 3 sub-cutaneous injections of 100 μl of the selected media at the dorsal region at 3-day intervals and were monitored for their food intake, body weight and any changes in the physical appearance, every alternate day, for a period of 20 days. Upon 20 days the mice were subjected to euthanasia. The organs skin, brain, heart, liver, lungs, kidney, spleen and pancreas of the euthanized mice were harvested. The tissues were embedded in paraffin wax and sectioned using the Leica Microtome longitudinally to obtain 3 μm sections. The sectioned tissues were stained using H&E staining. The slides were scanned using the digital scanning system (Pannoramic Desk). The cellular and tissue morphology were assessed by a pathologist to observe any abnormality.

## Results

### Morphology and growth kinetics of SHED and HFSCs cultured in different media combinations

**Fig 1:**
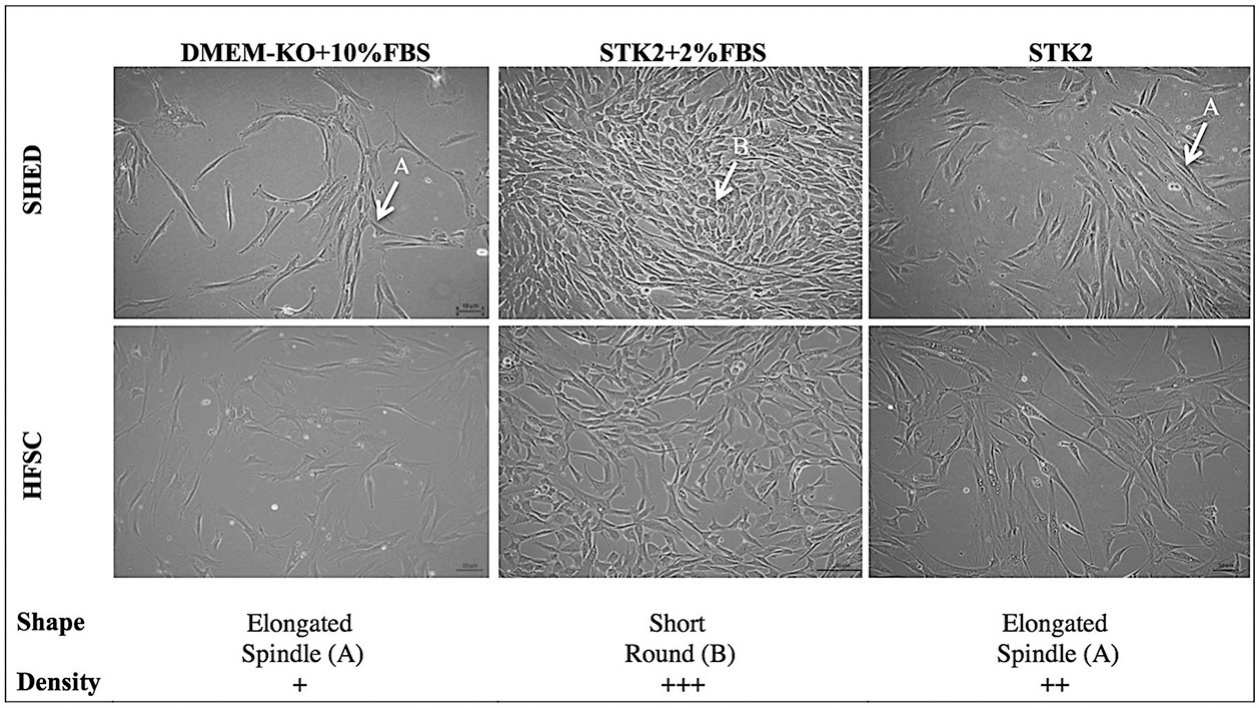
Morphology and cell density of SHED and HFSCs cultured in different media combinations. **The cell morphology of SHED and HFSCs in the media combinations;** DMEM-KO+10% FBS, STK2+2% FBS+STK2 at Day 3 of passage 3, observed under 10× magnification. *A- elongated spindle shape, B-short round shape, +-low cell density, ++- moderate cell density, +++- high cell density.

SHED and HFSCs were cultured from passage 2 to 5 in DMEM-KO+10% FBS, STK2+2% FBS and STK2 media. The cells exhibited a spindle shaped morphology when cultured in all media combinations (Fig 1). Based on the morphological assessment, the highest cell density was in STK2+2% FBS media, followed by STK2 serum-free media and the least density was in DMEM-KO+10% FBS at a given period.

**Fig 2:**
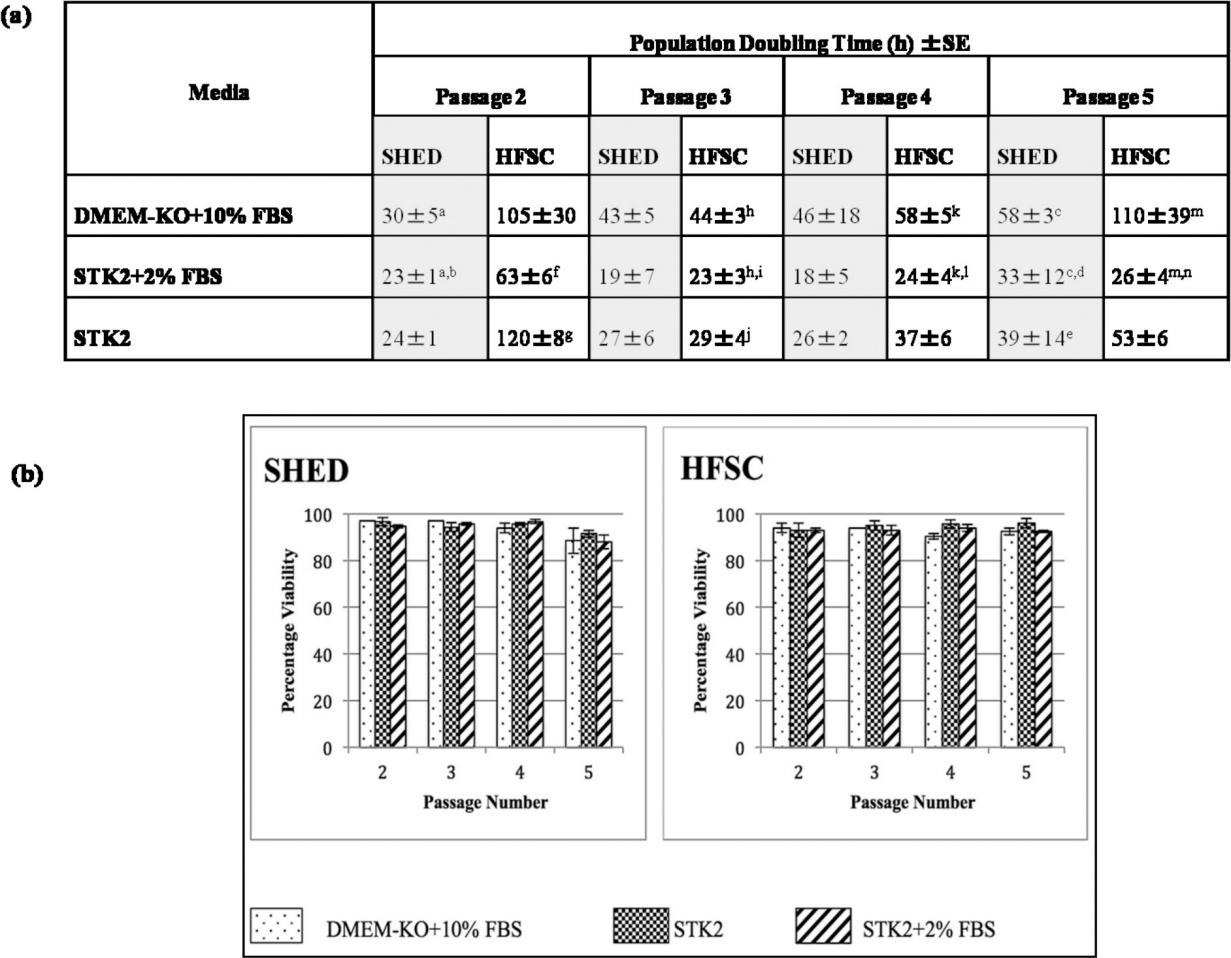
The population doubling times and viability of SHED and HFSCs cultured in the different media combinations from passage 2 to passage 5. (a) The population doubling times of SHED and HFSCs cultured in the different media combinations from passage 2 to passage 5. *The same alphabet indicates a significant difference (p<0.05). There was no statistically significant difference between the population doubling times between SHED and HFSC. (b)The percentage of viability of SHED and HFSCs cultured in media combinations; DMEM-KO+10% FBS, STK2+2% FBS and STK2 from passages 2 to 5.

The assessment of PDT indicated that SHED had a lower PDT ranging between 18–58 hrs than HFSCs which ranges between 23–120 hrs throughout the passages (Fig 2). A lower PDT is observed in both stem cell sources at passage 3 and 4 in comparison to the passage 5 indicating their proliferative capacity reduces along the passages. Interestingly, the PDT analysis showed that cells cultured in STK2 based media with or without serum has a lower PDT in comparison to DMEM-KO based media throughout all passages.

SHED cultured in STK2+2% FBS at passage 4, showed the lowest population doubling of 18±5 h. SHED cultured in STK2 based media showed significantly (p<0.05) lower PDT than DMEM-KO media at passages 2 and 5. HFSCs showed the lowest PDT when cultured in STK2+2% FBS media in passage 3 (23±3 h). The HFSCs cultured in the STK2 based media for all passages showed significantly (p< 0.05) lower PDT than DMEM-KO based media.

In order to assess the effect of these media combinations on the viability of stem cells, Tryphan blue exclusion method was used. It was found that the percentage of viability was higher in passages 3 and 4 in comparison to the passage 5 SHED and HFSCs. Cells cultured in STK2 based media showed a higher viability in all passages in comparison to the DMEM-KO based media. The highest viability was observed in passage 3 for SHED cultured in STK2+2% FBS (98%±0.001), and at passage 4 for HFSCs when cultured in STK2 (98%±0.007). However, no significant difference (p>0.05) was observed in viability between SHED and HFSCs or between the media combinations for each source (Fig 2).

### Characterization of SHED and HFSCs for their mesenchymal stem cell properties

#### Determination of the mesenchymal stem cell marker expression

99% of SHED cultured in STK2 based media was positive for CD90 marker, while 94% cells expressed CD90 when expanded in DMEM-KO based media. 95% of HFSCs expressed CD90 (S1 Table) when cultured in STK2 based media while 69% of HFSCs expressed CD90 when cultured in DMEM-KO based media. The positive markers CD105 and CD73 were expressed between 95–100% for both SHED and HFSCs in all media combinations (S2 Table). The results also indicated that a higher homogeneity of the HFSCs was maintained in the STK2 based media in comparison to DMEM-KO media.

In SHED, 13% of the cells expressed negative markers when cultured in STK2+2% FBS media. For all other media combinations, the negative marker expression was less than 7% of the cell population in both SHED and HFSCs.

#### Maintenance of differentiation potential of SHED and HFSCs

The adipogenic differentiation was assessed by staining cytoplasmic lipid droplets with Oil Red O stain. The staining of proteoglycans with Safranin confirmed the chondrogenic differentiation while the differentiation of the stem cells to osteogenic lineage was confirmed by staining of extracellular calcium deposits using Alzarin red. The result from the differentiation studies confirmed that all media combinations supported the maintenance of their stem cell characteristics in which these cells were able to commit to the respective mesenchymal lineages (S2 Fig and S3 Fig).

### Profiling of paracrine secretion of SHED and HFSCs

#### Hair growth related paracrine secretion profile of SHED and HFSCs

Cells cultured in all media combinations showed secretion of positive hair growth-regulatory factors; VEGF-A and HGF. The secretion of negative hair growth-regulatory factors is comparatively higher in SHED and HFSCs when cultured in DMEM-KO+10% FBS compared to STK2 media. SHED and HFSCs cultured in STK2 based media secreted only BDNF and IL-1 as negative hair growth-regulatory factors. The paracrine profiles of CM produced by SHED and HFSCs indicated that STK2 media did not facilitate the secretion of the positive hair growth-regulatory factor SDF-1, while STK2+2% FBS did not facilitate the secretion of the positive hair growth-regulatory factor PDGF-BB. It is also observed that HFSCs secreted a higher amount of positive hair growth-regulatory factors than SHED (Tables 1 and 2).

**Table 1:** The mean (±SE) hair regulatory paracrine factor concentrations in ng/10^6^cells for SHED cultured under the different culture media combinations; DMEM-KO+10% FBS, STK2+2% FBS and STK2 at passage 3 and passage 4.

Paracrine Factor	DMEM-KO+10% FBS		STK2+2% FBS		STK2	
	P3	P4	P3	P4	P3	P4
SDF-1^*^	11.80**±** 4.52	9.96**±**1.63	0.55**±**0.42	0.74**±** 0.74	nd	nd
HGF^*^	13.20**±** 6.57	11.30**±**5.77	2.42**±**1.52	0.08**±** 0.05	2.85**±** 2.63	0.13**±**0.07
VEGF-A^*^	44.10**±**20.70	39.80**±**2.33	104.00**±**49.40	27.90**±**9.67	39.80**±**30.60	9.36**±**2.78
PDGFBB^*^	0.45**±** 0.30	0.24**±**0.01	nd	nd	0.59**±** 0.59	0.39**±**0.39
IL-1α^**#**^	0.11**±** 0.08	0.04**±**0.03	0.02**±**0.02	nd	0.004**±**0.004	0.007**±**0.007
IL-1β^**#**^	0.14**±** 0.08	0.07**±**0.04	nd	nd	nd	nd
TGF-β^**#**^	0.84 **±** 0.45	0.53**±**0.28	nd	nd	nd	nd
bFGF^**#**^	1.36 **±** 0.85	0.73**±**0.37	nd	nd	nd	nd
TNF-α^**#**^	1.29 **±** 0.77	0.85**±**0.43	nd	nd	nd	nd
BDNF^**#**^	0.94 **±** 0.67	1.40 **±**1.25	0.61**±**0.59	1.01**±**0.99	0.008**±**0.008	0.007**±**0.007

* Positive hair regulatory factors

^#^ Negative hair regulatory factors

P Passage number

nd not detected

**Table 2:** The mean (±SE) hair regulatory paracrine factor concentrations in ng/10^6^cells for HFSCs cultured under the different culture media combinations; DMEM-KO+10% FBS, STK2+2% FBS and STK2 at passage 3 and passage 4.

Paracrine Factor	DMEM-KO+10% FBS		STK2+2% FBS		STK2	
	P3	P4	P3	P4	P3	P4
SDF-1^*^	34.70±10.20	34.70± 10.20	2.86± 2.86	2.42± 2.42	nd	nd
HGF^*^	57.54± 3.46	2.81± 2.60	39.50±17.20	66.10±33.50	9.41± 8.58	28.10±26.50
VEGF-A^*^	175.00±85.70	159.00±136.00	81.30±32.50	106.00±47.50	273.0±20.90	90.60±73.40
PDGF-BB^*^	0.79± 0.04	1.31± 1.31	nd	nd	1.28± 1.28	2.31± 2.31
IL-1α^**#**^	0.21± 0.08	0.22± 0.15	0.05±0.02	nd	0.05± 0.05	0.10± 0.10
IL-1β^**#**^	0.32± 0.18	0.32± 0.32	nd	nd	0.01± 0.01	0.01± 0.01
TGF-β^**#**^	1.05± 0.53	1.83± 1.83	nd	nd	nd	nd
bFGF^**#**^	2.23± 1.23	1.87± 1.87	nd	nd	nd	nd
TNF-α^**#**^	3.72± 2.21	3.39± 3.39	nd	nd	nd	nd
BDNF^**#**^	1.41± 1.05	1.97± 1.97	0.19±0.19	nd	0.02±0.02	0.03±0.03

* Positive hair regulatory factors

^#^ Negative hair regulatory factors

P Passage number

nd not detected

### Determination of the most suitable conditioned media to stimulate hair growth *in vitro*

**Fig 3:**
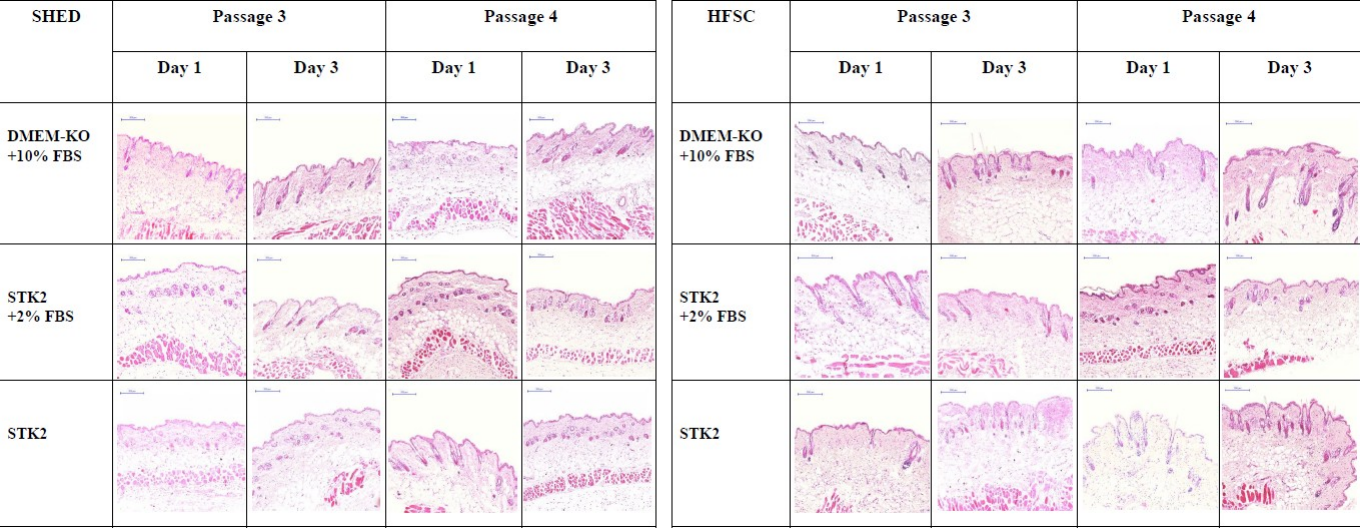
Representative photomicrographs of ICR mouse skin samples treated with SHED-CM and HFSC-CM. Original magnification 40×. Stained with H&E.

Following the paracrine profiling studies, *in vitro* hair growth stimulation for ICR mice skin was observed using SHED- and HFSC-CM to identify the most suitable CM to stimulate the hair growth. The kappa score for the inter-reliability tests for the tissue scoring was 0.8. The representative photomicrographs for hair follicles in early, mid and late anagen, catagen and telogen stages in the different media combinations are shown in Fig 3.

The *in vitro* study indicated the transition of hair follicles to subsequent hair growth stages following the synchronization of the hair follicles to telogen stage. Students paired t-test indicated no significant increase of the number of hair follicles by day 3 in the control media. DMEM-KO+10% FBS, STK2, STK2+2%FBS media showed a significant increase (p<0.05) in the number of mid anagen-staged hair follicles. STK2+2% FBS also showed a significant decrease in the number of telogen hair follicles in day 3.

**Fig 4:**
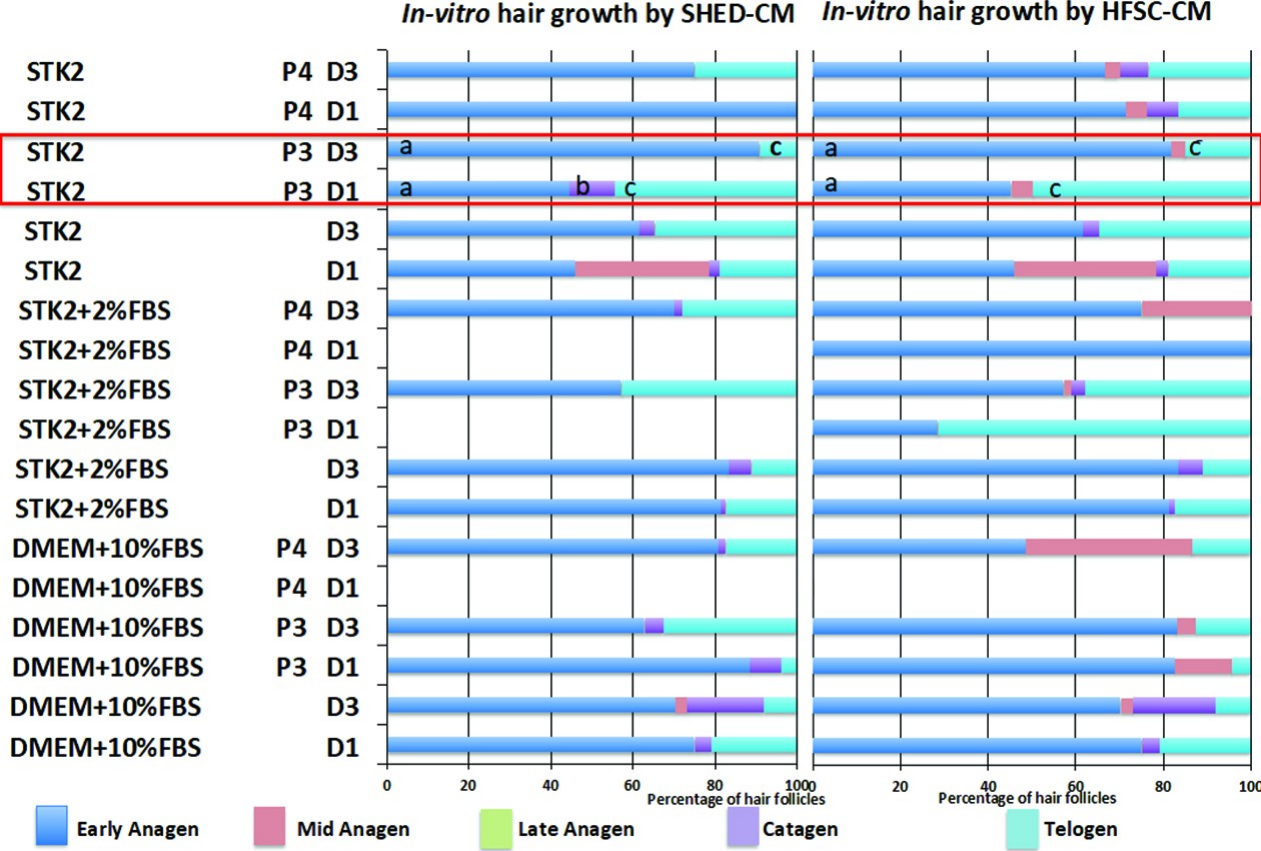
Percentage of hair follicles at each hair growth stage on day 1 and day 3, post CM treatment under *in vitro* conditions. The percentage of hair follicles transferred from the telogen-stage to the subsequent stages of hair growth following the CM treatment. *The same alphabet indicates a significant difference (p<0.05) aa-Significant increase in anagen stage bb-Significant decrease in catagen stage cc-significant decrease in telogen stage D = Day P = Passage.

It was observed that all the tissues treated with CM had a significant increase (p<0.05) in the early anagen stage hair follicles by day 3 in comparison to day 1, except for the CM obtained from HFSCs and SHED cultured in STK2 media at passage 4 (Fig 4). The mid anagen hair follicle number significantly increased (p<0.05) in HFSC-CM collected in cells expanded in DMEM-KO+10% FBS at passage 4 and STK2+2% FBS at passage 3 and 4. The number of catagen hair follicles were significantly increased (p<0.05) in HFSC-CM prepared by the expansion of cells in STK2+2% FBS at passage 3, SHED-CM prepared by the expansion of cells in DMEM-KO+10% FBS+ bFGF in passage 3, DMEM-KO+10% FBS passage 4, STK2+2% FBS in passage 4 making the media not suitable for the preparation for CM to stimulate hair growth. The number of hair follicles in telogen stage was significantly increased (p<0.05) by day 3 in SHED-CM and HFSC-CM collected in STK2+2% FBS at passage 3 and DMEM-KO+10% FBS in passage 4. This was also observed in SHED-CM collected by expansion of cells in STK2 and STK2+2% FBS in passage 4. Thus, due to the undesirable increase in the hair follicle numbers in telogen stage, we eliminated these media for further *in vivo* studies.

However, specimens treated with STK2 media at passage 3 indicated a significant increase (p<0.05) in anagen stage hair follicles and a significant decrease (p<0.05) in the catagen and telogen stage hair follicles with respect to SHED-CM, while the same media at passage 3 showed a significant increase (p<0.05) in the anagen stage hair follicles and a significant decrease (p<0.05) in the number of hair follicles telogen stage by day 3 for HFSC-CM. Based on the *in vitro* study results the STK2 media at passage 3 was chosen for subsequent *in vivo* study.

### Determination of the potential of conditioned media to stimulate hair growth under *in vivo* conditions

**Fig 5:**
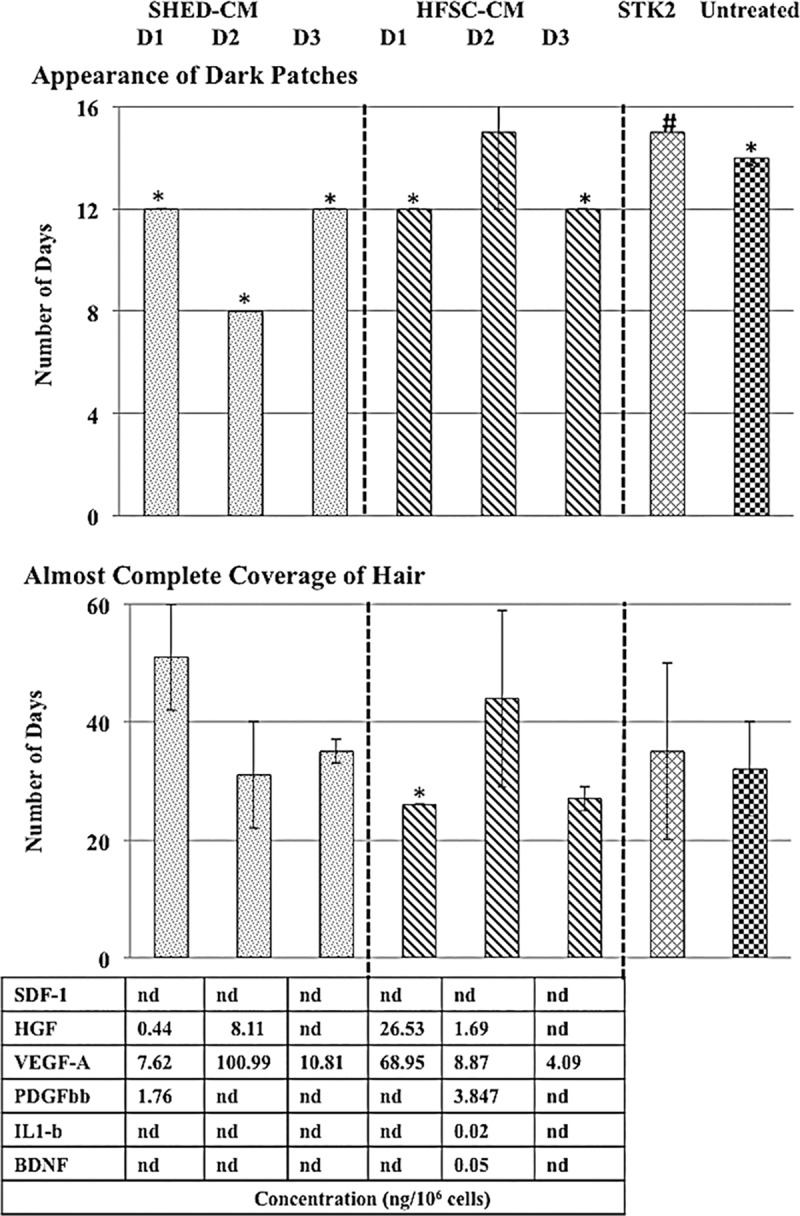
Number of days taken for the appearance of dark patches and almost complete hair coverage with the corresponding paracrine factor profiling of the CM prepared in STK2-serum free media. The number of days taken for the appearance of dark patches and almost complete hair coverage in C3H/HeN mice when treated with three subcutaneous injections of 100μl of SHED-CM (n = 3), HFSC-CM (n = 3) and STK2 (n = 3) at three day intervals and the untreated C3H/HeN mice (n = 2). The corresponding cytokine profiling for the donor from which the CM was prepared in also indicated. * Standard error = 0, # Standard error = 1 and D = Donor.

The *in vivo* study was conducted by injecting 3 sub-cutaneous injections of 100 μl of CM to telogen synchronized C3H/HeN mice at three-day intervals. Pictorial recording was made for each mouse at alternate days for the observation of the appearance of dark patches and until almost complete hair coverage of the skin was observed (S4 Fig). The visual observance for the appearance of dark patches indicating the transition from telogen to anagen stage by SHED-CM ranged from 8 to 12 days while HFSC-CM ranged from 12 to 15 days (Fig 5) post-administration of CM. The mice treated with STK2 showed the appearance of dark patches on day 15. This is longer than the average number of days taken for the appearance of dark patches, for SHED-CM (11 days) and HFSC-CM (13 days) treatments. For the untreated mice, dark patches appeared on day 14, higher than the mean number of days taken for those groups treated with SHED-CM and HFSC-CM. This demonstrates that in SHED-CM, the transition of telogen stage to anagen stage has occurred comparatively faster. However, the almost complete hair growth of the shaved skin area in SHED-CM ranged between 31 to 51 days, while HFSC-CM ranged between 26 to 44 days. The paracrine profiling carried out for the individual donors demonstrate that the CM which contained a higher VEGF–A and HGF level have shown a quicker transition from telogen to anagen stage (SHED-CM- Donor 2, HFSC-CM- Donor 1) (Fig 5).

**Fig 6:**
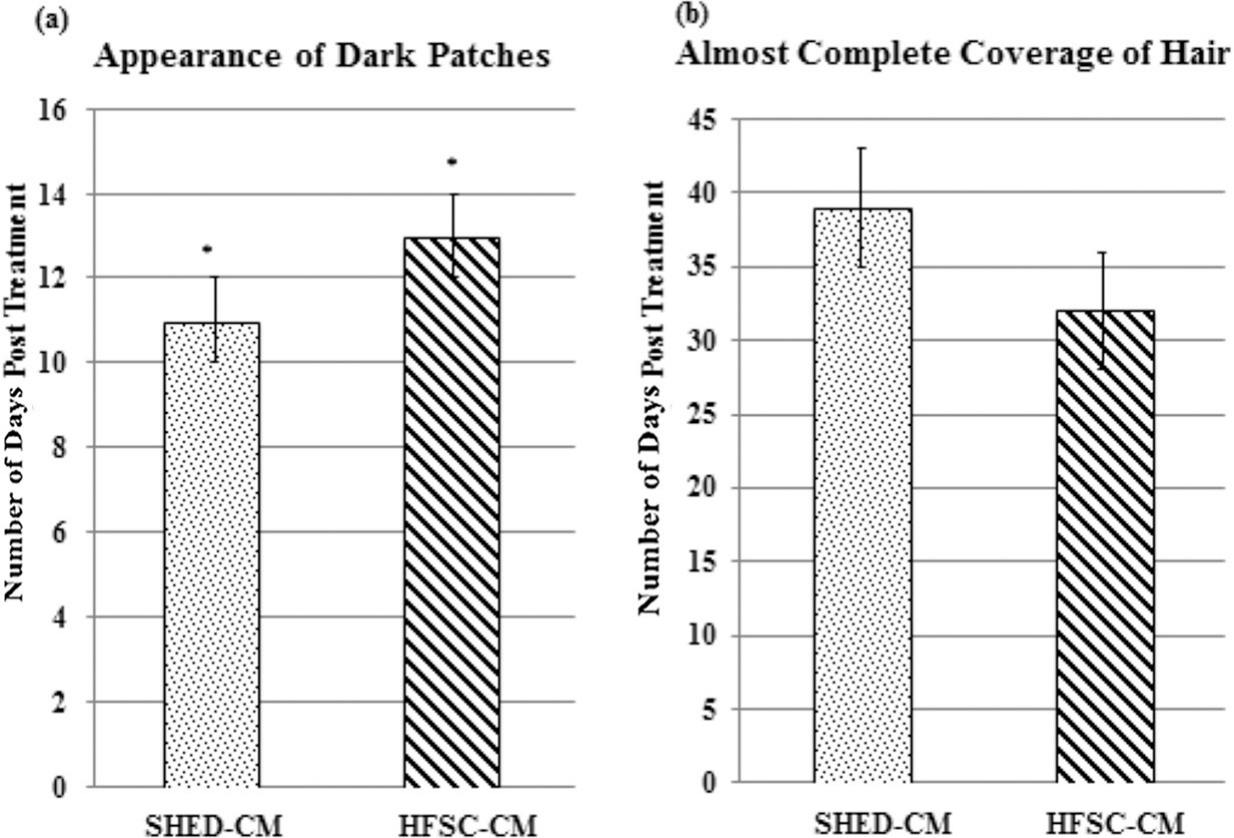
Time duration taken for the appearance of dark patches and almost complete coverage of hair upon treatment with SHED-CM and HFSC-CM. **(**a) The number of days taken for the appearance of dark patches in C3H/HeN mice (n = 9) when treated with three subcutaneous injections of 100 μl of SHED-CM and HFSC-CM at three day intervals (b) The number of days taken for almost complete hair coverage of the C3H/HeN mice (n = 9) when treated with three subcutaneous injections of 100μl of SHED-CM and HFSC-CM at three day intervals. * indicates a significant difference (p<0.05).

In order to compare the effect of the stem cell source in hair growth stimulation, statistical analysis was conducted using students paired t-test, combining the results for the SHED-CM and HFSC-CM (n = 9 per stem cell source). The appearance of dark patches was significantly faster (p<0.05) in SHED-CM in comparison to the HFSC-CM (Fig 6A). The almost complete hair coverage was observed earlier when treated by HFSC-CM (Fig 6B). However, there was no significant difference between SHED-CM and HFSC-CM with respect to the observation made related to the effect of the two sources of CM in reaching almost complete hair coverage.

The percentage of hair growth observed between day 7–14 (S5 Fig) also indicated that the stimulation of hair growth is faster in SHED-CM in comparison to the HFSC-CM. The HFSC-CM and the control groups (STK2 and untreated samples) only showed hair growth stimulation after day 12. It was observed that following 2 weeks of the CM administration, the HFSC-CM indicated the highest hair growth stimulation followed by SHED-CM, STK2 control media and untreated mice. However, 3 weeks post-treatment, hair growth in the untreated mice increased than the SHED-CM treated mice. The activity of HFSC-CM indicted to be declining following the 3^rd^ week in comparison to the SHED-CM. The STK2 media shows a higher percentage of hair growth stimulation than the untreated mice until week 3. However, at week 4 post–treatment the untreated mice groups showed the highest percentage of hair growth.

### *In vivo* toxicity studies of the conditioned media

During the period of observation the mice remained active and in good health condition. The histological analysis also showed that there was no change in the morphology of the tissues upon the administration of CM.

## Discussion

Stem cell derived CM is widely explored for its therapeutic potential in replacement to cell-based therapies for a wide range of diseases [26]. Thus, we aimed to evaluate the potential of CM from SHED to stimulate hair growth. The selection of culture media plays a vital role in this regard. The media should be able to facilitate the cells to secrete positive hair growth- regulatory factors and also help cells maintain the MSC properties.

Considering previous studies on culture media that enable both HFSCs and DPSCs cultures [27–29], we used DMEM-KO supplemented with 10% FBS to culture the cells. However, since xeno-free media has always been considered as an alternative to FBS supplemented media for cell-based therapy [30], serum-free STK2 media was also chosen for the expansion of cells. Even though STK2 media has been used to culture DPSCs, BMSCs, ADSCs and Synovial MSCs [31–33] to the best of our knowledge no attempt has been reported to culture HFSCs in this media.

The cells were cultured in the different media combinations; i) STK2, ii) DMEM-KO+10% FBS, iii) STK2+2% FBS. In all media combinations the cells showed fibroblast like, spindled-shaped morphology as earlier described for SHED [34] and HFSCs [35]. A higher cell density and a lower PDT was reported in SHED and HFSCs cultured in STK2 based media. Even though the PDT between the cell sources was not significantly different, SHED showed a better proliferative capacity than HFSCs. The flow cytometry and tri-lineage differentiation studies confirmed that the MSC properties of the cells were maintained in all media combinations.

The hair cycle is maintained by a series of cascade events controlled by paracrine factors. Thus, we profiled the main positive hair growth-regulatory factors SDF-1, HGF [16, 36–38], VEGF-A [39], PDGF-BB and negative hair growth-regulatory factors; IL-1α, IL-1β [40], TNF-α [41], TGF-β [42], bFGF[43], and BDNF [44] present in the CM.

During the profiling studies carried out, it was observed that the DMEM-KO based media seem to promote the secretion of all negative hair regulatory factors, while STK2 based media only influenced in the secretion of the negative hair growth-regulatory factors, IL-1α and BDNF. Studies conducted by Gazarian and Ramírez-García has shown the differential expression of genes when SHED is supplemented with different concentrations of FBS [45].

Considering the results obtained from the population doubling, viability, flow cytometry and trilineage differentiation studies, STK2 based media was more suitable for the expansion of SHED and HFSCs. This was further the media of choice to prepare CM, since STK2 based media did not promote the secretion of the negative hair growth-regulatory paracrine factors.

A short anagen stage followed by a synchronized entry of the hair follicles to the telogen stage is mainly observed in all forms of hair related diseases. An *in vitro* study was then conducted to evaluate the potential of CM to stimulate the hair follicles in telogen stage to enter anagen stage. No significant difference in the number of hair follicles entering the anagen stage was observed for tissues immersed in culture media that was not conditioned by the stem cells even after an incubation period of 72 h. An early onset of the catagen stage was observed in unconditioned STK2 and DMEM-KO media, SHED-CM prepared in STK2 at passage 3, STK2+2% FBS at passage 4 and DMEM-KO+10% FBS at passages 3 and 4, and HFSC-CM prepared in STK2 at passage 4 and STK2+2% FBS at passage 3.This observation may be due to the absence of insulin in the CM which is responsible in preventing the entry of hair follicles from anagen to catagen stage [46]. The *in vitro* studies confirmed that STK2 media in passage 3 as most suitable CM in the stimulating hair growth. This media significantly (p<0.05) increased the number of hair follicles in the anagen stage and significantly decreased (p<0.05) the number of hair follicles in the catagen and telogen stages.

Since the *in vitro* study only accounts for a partial sequence of events that occurred within a biological system, a preliminary *in vivo* study was conducted on seven-week-old C3H/HeN mice by injecting CM prepared from STK2 when SHED and HFSCs were cultured at passage 3. However, the mice models only represent hair growth under physiological conditions, which remains as a limitation of the study. Muller-Rover (2001), Park (2008) and Won (2010) have reported that the transition of telogen-staged hair follicles to the anagen stage is depicted by the appearance of dark patches of seven-week-old mice upon shaving [25,47–48]. The appearance of dark patches in the C3H/HeN mice treated with SHED-CM were observed within 11±2 days post-treatment while HFSC-CM demonstrated appearance of dark patches within 13±2 days. Our results concurred with Muller Rover (2010) who showed that the anagen to telogen stage of hair transition occurs within 25 days. The increased paracrine factor gradient in the mouse physiological system would have resulted in the stimulation of hair growth. It is likely that the increase in paracrine gradient caused the HFSCs in the niche to proliferate and differentiate and increase vascularization in the treated area [49–50].

All mice groups showed almost complete hair coverage between days 31 to 39. Although mice treated with SHED-CM showed the longest duration to reach almost complete hair coverage, the difference was not significant (p>0.05) to HFSC-CM and the degradation of paracrine factors occurs in several distinct pathways such as proteolysis, oxidation and denaturation [51] within the biological system. This results in the reduction of paracrine gradient with time [52], which in turn reduces the efficiency of the administered CM [53]. It is noteworthy that a stable paracrine factor gradient is required in order to maintain the stimulated hair growth [54]. Further studies should be conducted to develop suitable delivery systems that would maintain the stability of the pool of paracrine factors that are present in the CM [4, 55]. Furthermore, the increase of the frequency of CM application, optimization of an efficient dosage and establishment of suitable delivery methods [51] should be explored to improve the long-term desirable therapeutic outcome.

Our preliminary study suggests that SHED-CM prepared in STK2 media has the potential of stimulating hair growth. However, employing different culture methods such as 3D floating sphere cultures may provide the opportunity of enriching the CM with more positive hair regulatory paracrine factors [56].Since SHED is isolated from extracted or exfoliated teeth, which could be considered as a waste product, it acts as a non-controversial source, thus drawing less ethical concerns [19]. It also has advantages with respect to its safety, minimal invasiveness and discomfort caused to the donors during the process of harvesting of stem cells from this source. Furthermore, SHED can be obtained from young donors and stored for future usage [57]. In conclusion, within the limitations of the preliminary *in vivo* study we form a strong foundation to necessitate the exploration of SHED-CM as a potential therapeutic tool for hair loss.

## Supporting information

S1 Fig Photomicrographs to indicate the different stages of hair growth. (a) Score chart for the determination of murine hair growth stage adapted from Muller Rover et al. 2001. (b) The representative photomicrographs of the hair follicle indicating the different hair growth stages, identified based on the position of the hair follicle, consistency and shape of the dermal papilla and the presence and absence of the inner root sheath (Original Magnification 40× and stained with H&E staining).
(PDF)

S2 Fig Tri-lineage differentiation of SHED. The trilineage differentiation studies conducted to study the maintenance of MSC lineages; adipogenic, chondrogenic and osteogenic for SHED when cultured in media combinations; DMEM-KO+10% FBS, STK2+2% FBS and STK2.The representative images of cells cultured in DMEM-KO+10% FBS as the control media. The study was carried out for the cells at passage 3 upon 80% confluency.
(PDF)

S3 Fig Tri-lineage differentiation of HFSCs. The trilineage differentiation studies conducted to study the maintenance of MSC lineages; adipogenic, chondrogenic and osteogenic for SHED and HFSCs when cultured in media combinations; DMEM-KO+10% FBS, STK2+2% FBS and STK2.The representative images of cells cultured in DMEM-KO+10% FBS as the control media. The study was carried out for the cells at passage 3 upon 80% confluency.
(PDF)

S4 Fig Pictorial representation for the appearance of dark patches and almost complete coverage with newly grown hair. The photographs of the telogen synchronized 7 week old female C3H/HeN mice following the subcutaneous injection of 100μl of SHED-CM (n = 9) and HFSC-CM (n = 9) administered at three day intervals for three days, for the observation of dark patches and almost complete coverage with newly grown hair.
(PDF)

S5 Fig Percentage indication of hair growth. (a) The percentage of hair growth from Day 7- Day 14, following three subcutaneous injections of 100 μl of SHED-CM (n = 9), HFSC-CM (n = 9), STK2 (n = 3) at three-day intervals to the C3H/HeN mice and the percentage indication of hair growth for the untreated C3H/HeN mice (n = 2) (b)Weekly progress of the percentage of hair growth following three subcutaneous injections of 100 μl of SHED-CM (n = 9), HFSC-CM (n = 9), STK2 (n = 3) at three-day intervals to the C3H/HeN mice and the percentage of hair growth for the untreated C3H/HeN mice (n = 2)
(PDF)

S1 Table Flowcytometry analysis of SHED. The positive and negative MSC marker expression of SHED when cultured in media combinations; DMEM-KO+10% FBS, STK2+2% FBS and STK2. The analysis was carried out for the cells at passage 3 upon 80% confluency.
(PDF)

S2 Table Flowcytometry analysis of HFSCs. The positive and negative MSC marker expression of HFSCs when cultured in media combinations; DMEM-KO+10% FBS, STK2+2% FBS and STK2. The analysis was carried out for the cells at passage 3 upon 80% confluency.
(PDF)

S1 Dataset Data sets used to reach the conclusions drawn in the manuscript. (PDF)
