# Supplementary material for: Dental derived stem cell conditioned media for hair growth stimulation
Source: PLoS One. 2019 May 1;14(5):e0216003. doi: 10.1371/journal.pone.0216003 (PMC6493760; doi:10.1371/journal.pone.0216003)
Supplement: S1 Fig — (a) Score chart for the determination of murine hair growth stage adapted from Muller Rover et al. 2001. (b) The representative photomicrographs of the hair follicle indicating the different hair growth stages, identified based on the position of the hair follicle, consistency and shape of the dermal papilla and the presence and absence of the inner root sheath (Original Magnification 40× and stained with H&E staining). (PDF) [file pone.0216003.s001.pdf]

(a)	Position of Hair Follicle					Consistency of Dermal Papilla		Shape of Dermal Papilla			Inner Root Sheath				
											Present			Absent	
	Papillary Dermis	Reticular Dermis	Border of Dermis and Subcutaneous	Mid Sub-Cutaneous	Deep SC above Panniculus Carnosus	Consistent	Diffused	Ball	Onion	Narrow	Cone	Bellow Sebaceous Gland	At Sebaceous Gland	Above Germ Cell	Bellow Germ Cell
Early Anagen															
Mid Anagen															
Late Anagen															
Catagen															
Telogen															

(b) **Early Anagen** | **Mid Anagen** | **Catagen** | **Telogen**

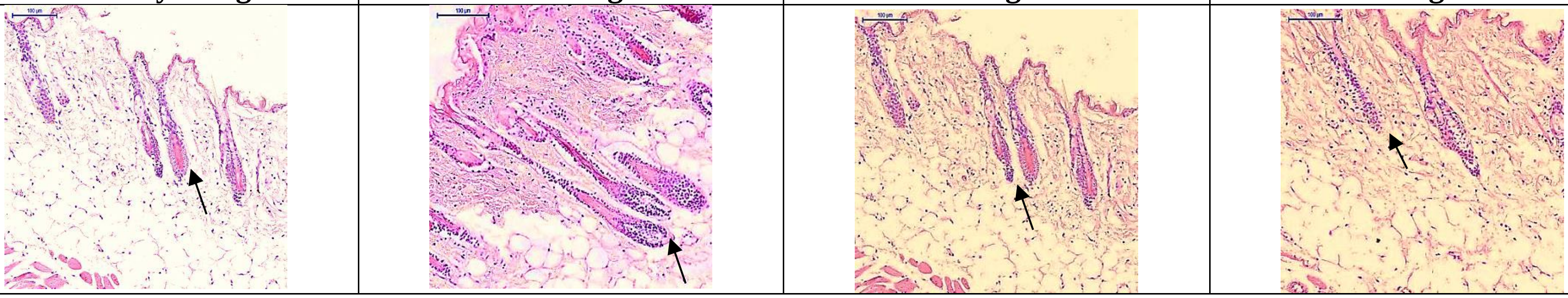

S1 Fig. Photomicrographs to indicate the different stages of hair growth (a) Score chart for the determination of murine hair growth stage adapted from Muller Rover et al. 2001. (b) The representative photomicrographs of the hair follicle indicating the different hair growth stages, identified based on the position of the hair follicle, consistency and shape of the dermal papilla and the presence and absence of the inner root sheath (Original Magnification 40× and stained with H&E staining)
