# Supplementary material for: Dental derived stem cell conditioned media for hair growth stimulation
Source: PLoS One. 2019 May 1;14(5):e0216003. doi: 10.1371/journal.pone.0216003 (PMC6493760; doi:10.1371/journal.pone.0216003)
Supplement: S2 Fig — The trilineage differentiation studies conducted to study the maintenance of MSC lineages; adipogenic, chondrogenic and osteogenic for SHED when cultured in media combinations; DMEM-KO+10% FBS, STK2+2% FBS and STK2.The representative images of cells cultured in DMEM-KO+10% FBS as the control media. The study was carried out for the cells at passage 3 upon 80% confluency. (PDF) [file pone.0216003.s002.pdf]

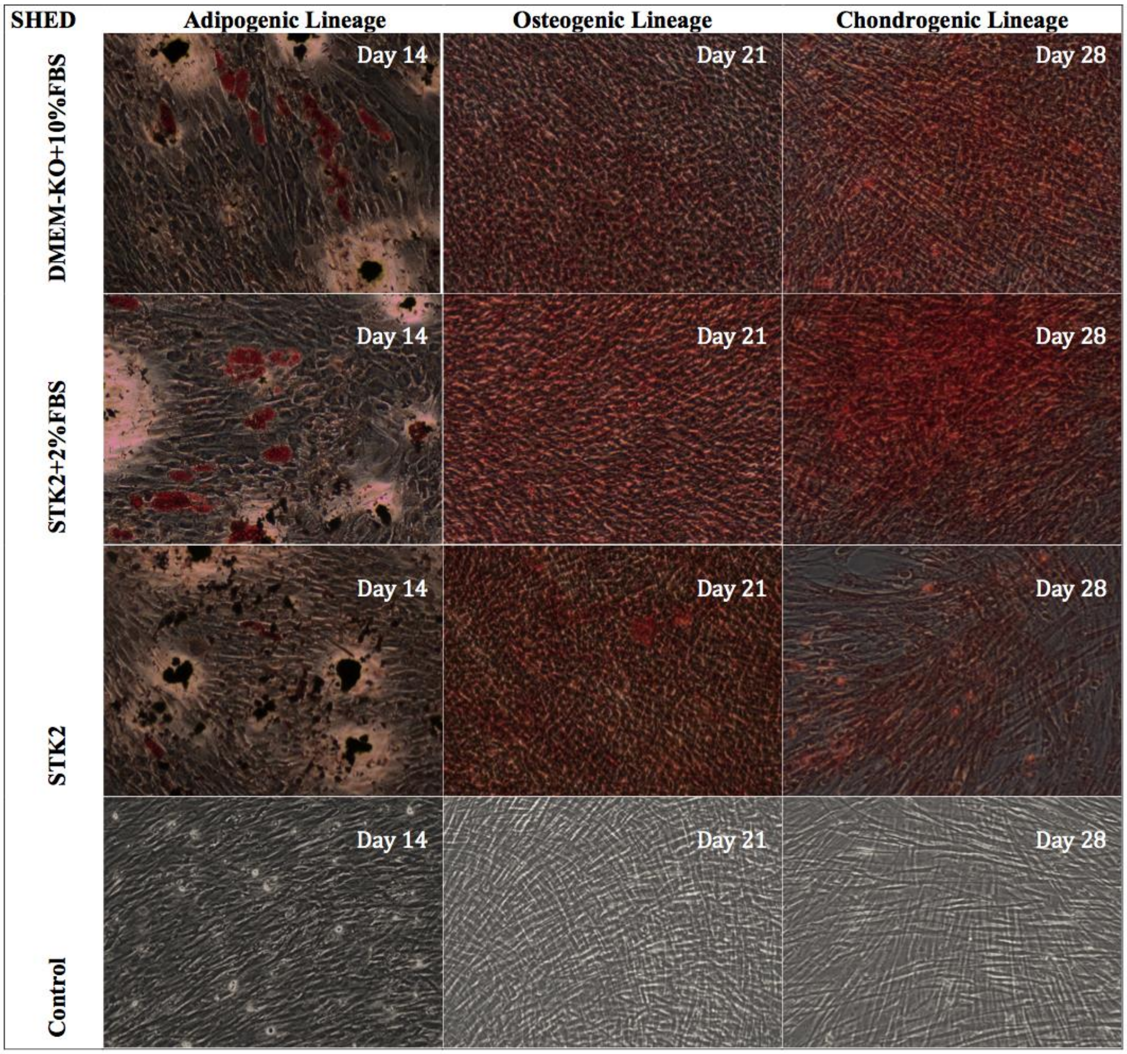

S2 Fig Tri-lineage differentiation of SHED. The trilineage differentiation studies conducted to study the maintenance of MSC lineages; adipogenic, chondrogenic and osteogenic for SHED when cultured in media combinations; DMEM-KO+10% FBS, STK2+2% FBS and STK2.The representative images of cells cultured in DMEM-KO+10% FBS as the control media. The study was carried out for the cells at passage 3 upon 80% confluency
