# Supplementary material for: Dental derived stem cell conditioned media for hair growth stimulation
Source: PLoS One. 2019 May 1;14(5):e0216003. doi: 10.1371/journal.pone.0216003 (PMC6493760; doi:10.1371/journal.pone.0216003)
Supplement: S4 Fig — The photographs of the telogen synchronized 7 week old female C3H/HeN mice following the subcutaneous injection of 100μl of SHED-CM (n = 9) and HFSC-CM (n = 9) administered at three day intervals for three days, for the observation of dark patches and almost complete coverage with newly grown hair. (PDF) [file pone.0216003.s004.pdf]

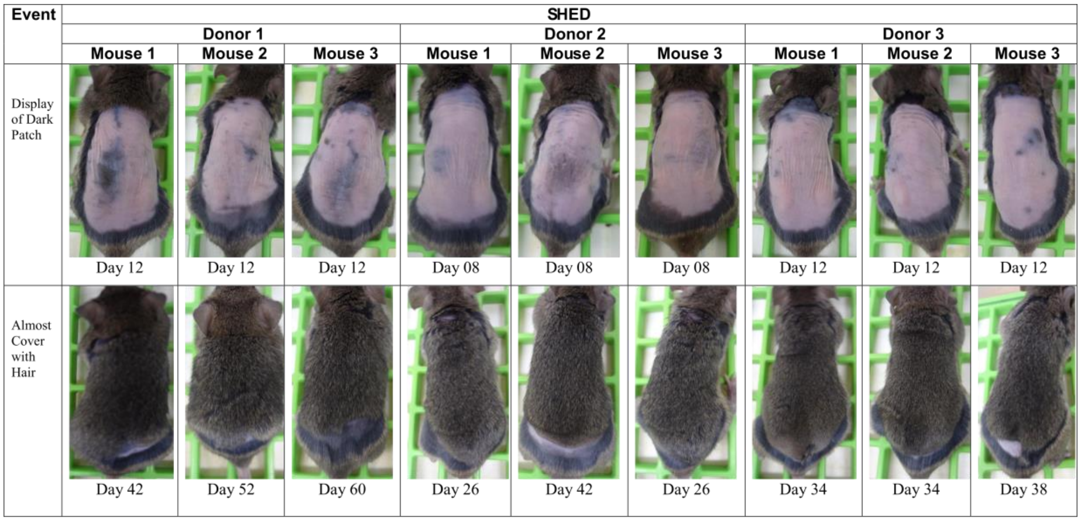

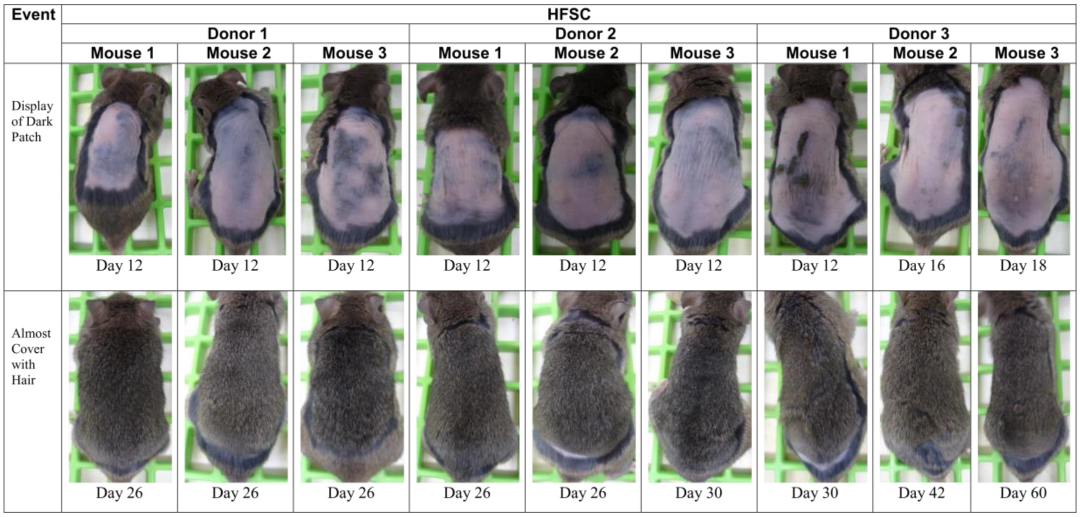

S4 Fig Pictorial representation for the appearance of dark patches and almost complete coverage with newly grown hair. The photographs of the telogen synchronized 7 week old female C3H/HeN mice following the subcutaneous injection of 100μl of SHED-CM (n=9) and HFSC-CM (n=9) administered at three day intervals for three days, for the observation of dark patches and almost complete coverage with newly grown hair
