# Supplementary material for: Dental derived stem cell conditioned media for hair growth stimulation
Source: PLoS One. 2019 May 1;14(5):e0216003. doi: 10.1371/journal.pone.0216003 (PMC6493760; doi:10.1371/journal.pone.0216003)
Supplement: S5 Fig — (a) The percentage of hair growth from Day 7- Day 14, following three subcutaneous injections of 100 μl of SHED-CM (n = 9), HFSC-CM (n = 9), STK2 (n = 3) at three-day intervals to the C3H/HeN mice and the percentage indication of hair growth for the untreated C3H/HeN mice (n = 2) (b)Weekly progress of the percentage of hair growth following three subcutaneous injections of 100 μl of SHED-CM (n = 9), HFSC-CM (n = 9), STK2 (n = 3) at three-day intervals to the C3H/HeN mice and the percentage of hair growth for the untreated C3H/HeN mice (n = 2) (PDF) [file pone.0216003.s005.pdf]

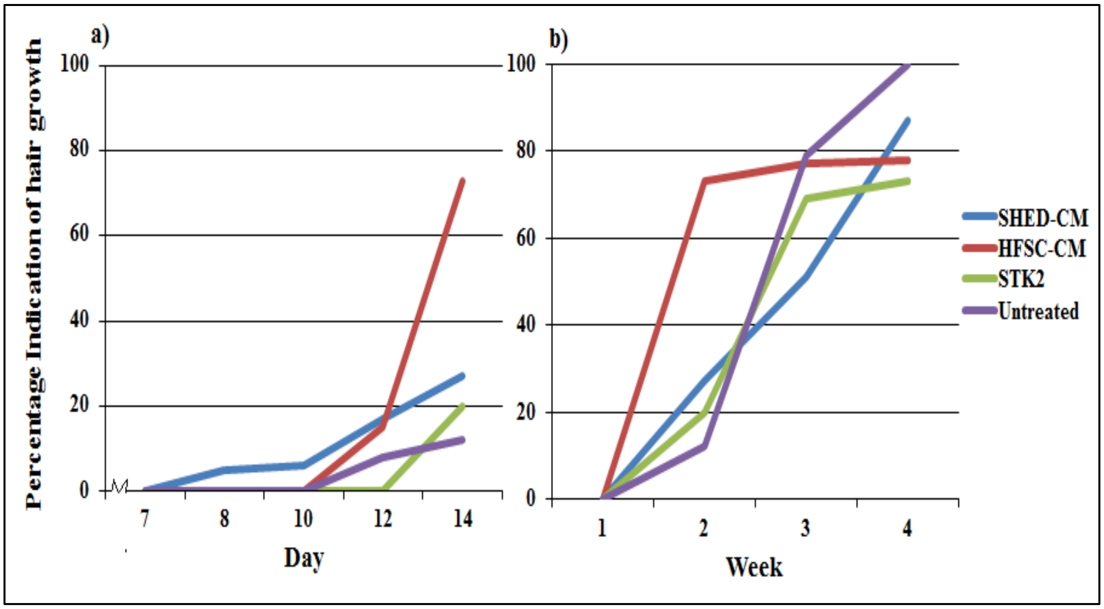

S5 Fig Percentage indication of hair growth. (a) The percentage of hair growth from Day 7-Day 14, following three subcutaneous injections of 100 µl of SHED-CM (n=9), HFSC-CM (n=9), STK2 (n=3) at three-day intervals to the C3H/HeN mice and the percentage indication of hair growth for the untreated C3H/HeN mice (n=2) (b)Weekly progress of the percentage of hair growth following three subcutaneous injections of 100 µl of SHED-CM (n=9), HFSC-CM (n=9), STK2 (n=3) at three-day intervals to the C3H/HeN mice and the percentage of hair growth for the untreated C3H/HeN mice (n=2)
