# Supplementary material for: Dental derived stem cell conditioned media for hair growth stimulation
Source: PLoS One. 2019 May 1;14(5):e0216003. doi: 10.1371/journal.pone.0216003 (PMC6493760; doi:10.1371/journal.pone.0216003)
Supplement: S1 Table — The positive and negative MSC marker expression of SHED when cultured in media combinations; DMEM-KO+10% FBS, STK2+2% FBS and STK2. The analysis was carried out for the cells at passage 3 upon 80% confluency. (PDF) [file pone.0216003.s006.pdf]

**Media**	**Positive markers**			**Negative marker cocktail**
	**CD 90**	**CD 105**	**CD 73**	**CD 45, CD 34, CD14, CD 20**
DMEM-KO+10% FBS	94.40%	97.95%	99.85%	4.79%
STK2+2% FBS	99.20%	99.60%	99.87%	12.82%
STK2	99.09%	99.28%	99.71%	6.19%

S1 Table Flowcytometry analysis of SHED. The positive and negative MSC marker expression of SHED when cultured in media combinations; DMEM-KO+10% FBS, STK2+2% FBS and STK2. The analysis was carried out for the cells at passage 3 upon 80% confluency. The cells were stained by MSC Phenotyping cocktail, Human (MACS, Miltenyi Biotech)
