# Supplementary material for: Dental derived stem cell conditioned media for hair growth stimulation
Source: PLoS One. 2019 May 1;14(5):e0216003. doi: 10.1371/journal.pone.0216003 (PMC6493760; doi:10.1371/journal.pone.0216003)
Supplement: S2 Table — The positive and negative MSC marker expression of HFSCs when cultured in media combinations; DMEM-KO+10% FBS, STK2+2% FBS and STK2. The analysis was carried out for the cells at passage 3 upon 80% confluency. (PDF) [file pone.0216003.s007.pdf]

Media	Positive markers			Negative marker cocktail
	**CD 90**	**CD 105**	**CD 73**	**CD 45, CD 34, CD14 , CD 20**
DMEM-KO+10% FBS	69.44%	99.02%	98.68%	6.03%
STK2+2% FBS	94.52%	99.67%	99.75%	3.00%
STK2	94.71%	98.26%	99.53%	0.83%

S2 Table Flowcytometry analysis of HFSCs. The positive and negative MSC marker expression of HFSCs when cultured in media combinations; DMEM-KO+10% FBS, STK2+2% FBS and STK2. The analysis was carried out for the cells at passage 3 upon 80% confluency. The cells were stained by MSC Phenotyping cocktail, Human (MACS, Miltenyi Biotech)
