# Supplementary material for: Dental derived stem cell conditioned media for hair growth stimulation
Source: PLoS One. 2019 May 1;14(5):e0216003. doi: 10.1371/journal.pone.0216003 (PMC6493760; doi:10.1371/journal.pone.0216003)
Supplement: S1 Dataset — (PDF) [file pone.0216003.s008.pdf]

S1 Dataset. Data sets used to reach the conclusions drawn in the manuscript.

Fig 2: The population doubling times and viability of SHED and HFSCs cultured in the different media combinations from passage 2 to passage 5

HFSC	PDT (Hours)	
**Passage 2**	**Average**	**SE**
DMEM-KO+10%FBS	105	30
STK2+2%FBS	63	6
STK2	120	8
**Passage 3**	**Average**	**SE**
DMEM-KO+10%FBS	44	3
STK2+2%FBS	23	3
STK2	29	4
**Passage 4**	**Average**	**SE**
DMEM-KO+10%FBS	58	5
STK2+2%FBS	24	4
STK2	37	6
**Passage 5**	**Average**	**SE**
DMEM-KO+10%FBS	110	39
STK2+2%FBS	26	4
STK2	53	6

SHED	PDT(Hours)	
**Passage 2**	**Average**	**SE**
DMEM-KO+10%FBS	30	5
STK2+2%FBS	23	1
STK2	24	1
**Passage 3**	**Average**	**SE**
DMEM-KO+10%FBS	43	5
STK2+2%FBS	19	7
STK2	27	6
**Passage 4**	**Average**	**SE**
DMEM-KO+10%FBS	46	18
STK2+2%FBS	18	5
STK2	26	2
**Passage 5**	**Average**	**SE**
DMEM-KO+10%FBS	58	3
STK2+2%FBS	33	12
STK2	39	14

HFSC	Percentage Viability	
**Passage 2**	**Average**	**SE**
DMEM-KO+10%FBS	94	2
STK2+2%FBS	93	3
STK2	93	1
**Passage 3**	**Average**	**SE**
DMEM-KO+10%FBS	94	0
STK2+2%FBS	95	2
STK2	93	2
**Passage 4**	**Average**	**SE**
DMEM-KO+10%FBS	90	1
STK2+2%FBS	96	2
STK2	94	2
**Passage 5**	**Average**	**SE**
DMEM-KO+10%FBS	93	2
STK2+2%FBS	96	2
STK2	93	1

SHED	Percentage Viability	
**Passage 2**	**Average**	**SE**
DMEM-KO+10%FBS	97	0
STK2+2%FBS	97	2
STK2	95	1
**Passage 3**	**Average**	**SE**
DMEM-KO+10%FBS	97	0
STK2+2%FBS	94	2
STK2	96	1
**Passage 4**	**Average**	**SE**
DMEM-KO+10%FBS	94	2
STK2+2%FBS	96	1
STK2	97	1
**Passage 5**	**Average**	**SE**
DMEM-KO+10%FBS	89	6
STK2+2%FBS	92	2
STK2	88	3

Fig 4 Percentage of hair follicles at each hair growth stage on day 1 and day 3, post CM treatment under in vitro conditions

Perecntage of HFs in different growth stages

SHED_CM	Early Anagen	Mid Anagen	Late Anagen	Catogen	Telogen
DMEM+10%FBS D1	75	0	0	4	21
DMEM+10%FBS D3	70	3	0	19	8
DMEM+10%FBS P3 D1	88	0	0	8	4
DMEM+10%FBS P3 D3	63	0	0	5	33
DMEM+10%FBS P4 D1	0	0	0	0	0
DMEM+10%FBS P4 D3	81	0	0	2	17
STK2+2%FBS D1	81	0	0	1	17
STK2+2%FBS D3	83	0	0	6	11
STK2+2%FBS P3 D1	0	0	0	0	0
STK2+2%FBS P3 D3	57	0	0	0	43
STK2+2%FBS P4 D1	0	0	0	0	0
STK2+2%FBS P4 D3	70	0	0	2	28
STK2 D1	46	32	0	3	19
STK2 D3	62	0	0	4	35
STK2 P3 D1	44	0	0	11	44
STK2 P3 D3	91	0	0	0	9
STK2 P4 D1	100	0	0	0	0
STK2 P4 D3	75	0	0	0	25

Perecntage of HFs in different growth stages

HFSC_CM	Early Anagen	Mid Anagen	Late Anagen	Catogen	Telogen
DMEM+10%FBS D1	75	0	0	4	21
DMEM+10%FBS D3	70	3	0	19	8
DMEM+10%FBS P3 D1	83	13	0	0	4
DMEM+10%FBS P3 D3	83	4	0	0	13
DMEM+10%FBS P4 D1	0	0	0	0	0
DMEM+10%FBS P4 D3	49	38	0	0	14
STK2+2%FBS D1	81	0	0	1	17
STK2+2%FBS D3	83	0	0	6	11
STK2+2%FBS P3 D1	29	0	0	0	71
STK2+2%FBS P3 D3	57	2	0	3	38
STK2+2%FBS P4 D1	100	0	0	0	0
STK2+2%FBS P4 D3	75	25	0	0	0
STK2 D1	46	32	0	3	19

**STK2**	**D3**	62	0	0	4	35
**STK2**	**P3 D1**	45	5	0	0	50
**STK2**	**P3 D3**	82	3	0	0	15
**STK2**	**P4 D1**	71	5	0	7	17
**STK2**	**P4 D3**	67	3	0	7	23

Fig 5 Number of days taken for the appearance of dark patches and almost complete hair coverage with the corresponding paracrine factor profiling of the CM prepared in STK2-serum free media.

	Appearance of Dark Patches		Almost Complete coverage of hair growth	
	Average No of Days	SD	Average Days	SD
**Donor 1 SHED-CM**	12	0	51	9
**Donor 2 SHED-CM**	8	0	31	9
**Donor 3 SHED-CM**	12	0	35	2
**Donor 1 HFSC-CM**	12	0	26	0
**Donor 2 HFSC-CM**	15	3	44	15
**Donor 3 HFSC-CM**	12	0	27	2
**STK2**	15	1	35	15
**Untreated**	14	0	32	8

Fig 6 Time duration taken for the appearance of dark patches and almost complete coverage of hair upon treatment with SHED-CM and HFSC-CM

	Appearance of Dark Patches		Almost Complete coverage of hair growth	
	Day	SD	Day	SD
**SHED**	11	2	39	11
**HFSC**	13	2	32	12

S5 Fig Percentage indication of hair growth.

SHED-CM	Total area	Clear area	Dark patched area	Area with hair	% Clear area	% Dark patched area	% Hair area	% Indication of hair growth
**Day 7**		Completely clear						0%
**Day 8**	4195221	3995899	199323	0	95%	5%	0%	5%
**Day 10**	4344233	4063567	214627	66039	94%	5%	2%	6%
**Day 12**	4926098	4173279	481843	332712	85%	10%	7%	17%
**Week 2**	6640213	4969778	1001713	823187	75%	15%	12%	27%
**Week 3**	8336310	4046432	1785502	2504376	49%	21%	30%	51%
**Week 4**	7470538	971670	1576492	4922376	13%	21%	66%	87%

HFSC-CM	Total area	Clear area	Dark patched area	Area with hair	% Clear area	% Dark patched area	% Hair area	% Indication of hair growth
**Day 7**		Completely clear						0%
**Day 8**		Completely clear						0%
**Day 10**		Completely clear						0%
**Day 12**	4998570	3248198	637528	115242	65%	13%	2%	15%
**Week 2**	11844828	3208479	7916440	719507	27%	67%	6%	73%
**Week 3**	8353515	1945396	982169	5425950	23%	12%	65%	77%
**Week 4**	5238832	1140177	67990	4030665	22%	1%	77%	78%

STK2	Total area	Clear area	Dark patched area	Area with hair	% Clear area	% Dark patched area	% Hair area	% Indication of hair growth
**Day 7**		Completely clear						0%
**Day 8**		Completely clear						0%
**Day 10**		Completely clear						0%
**Day 12**		Completely clear						0%
**Week 2**	5194099	4139344	296345	757891	80%	6%	15%	20%
**Week 3**	7930445	2436514	1844873	3634205	31%	23%	46%	69%
**Week 4**	6792523	1848720	0	4928950	27%	0%	73%	73%

Untreated	Total area	Clear area	Dark patched area	Area with hair	% Clear area	% Dark patched area	% Hair area	% Indication of hair growth
**Day 7**		Completely clear						0%
**Day 8**		Completely clear						0%
**Day 10**		Completely clear						0%
**Day 12**	3353500	3097629	180995	74876	92%	5%	2%	8%
**Week 2**	4999061	4418673	159493	420895	88%	3%	8%	12%
**Week 3**	4957191	1056280	1851420	2049491	21%	37%	41%	79%
